# Supplementary material for: Hybrid Models and Biological Model Reduction with PyDSTool
Source: PLoS Comput Biol. 2012 Aug 9;8(8):e1002628. doi: 10.1371/journal.pcbi.1002628 (PMC3415397; doi:10.1371/journal.pcbi.1002628)
Supplement: Text S4 — Complete source code for the PyDSTool package (version 0.88.120504). Includes API documentation and help files linking to web pages. This file is identical to the current public release on Sourceforge.net. (ZIP) [file pcbi.1002628.s004.zip › PyDSTool/html/PyDSTool.Toolbox.optimizers.criterion.information_criteria.ModifiedAICCriterion-class.html]

xml version="1.0" encoding="ascii"?


PyDSTool.Toolbox.optimizers.criterion.information\_criteria.ModifiedAICCriterion


| Home | Trees | Indices | Help | PyDSTool |
| --- | --- | --- | --- | --- |

Package PyDSTool ::
Package Toolbox ::
Package optimizers ::
Package criterion ::
Module information\_criteria ::
Class ModifiedAICCriterion

[hide private]

[frames] | no frames]

# Class ModifiedAICCriterion

source code

```
object --+
         |
        ModifiedAICCriterion
```

---

The Akaike information criterion with several trials authorized


|  |  |
| --- | --- |
| Instance Methods | [hide private] |

|  |  |
| --- | --- |
| \_\_init\_\_(self, ftol, iterations\_max, correct\_factor=1.0, trials=5)  Initializes the criterion with a max number of iterations and an error fraction for the monotony test... | source code |

|  |  |
| --- | --- |
| \_\_call\_\_(self, state, \*\*kwargs)  Computes the stopping criterion | source code |

**Inherited from `object`**:
`__delattr__`,
`__getattribute__`,
`__hash__`,
`__new__`,
`__reduce__`,
`__reduce_ex__`,
`__repr__`,
`__setattr__`,
`__str__`


|  |  |
| --- | --- |
| Properties | [hide private] |

**Inherited from `object`**:
`__class__`


|  |  |
| --- | --- |
| Method Details | [hide private] |

|  |  |
| --- | --- |
| \_\_init\_\_(self, ftol, iterations\_max, correct\_factor=1.0, trials=5)  *(Constructor)* | source code |

```
Initializes the criterion with a max number of iterations and an error fraction for the monotony test
- ftol is the relative tolerance of the AIC criterion
- trials indicates how many time the criterion will return false when in fact the criterion was true
- correct_factor is the modifiying factor for the weight of the parameters size
```

Overrides: object.\_\_init\_\_

  


| Home | Trees | Indices | Help | PyDSTool |
| --- | --- | --- | --- | --- |

|  |  |
| --- | --- |
| Generated by Epydoc 3.0.1 on Fri May 4 15:24:09 2012 | http://epydoc.sourceforge.net |
